# Levels and Patterns of Nucleotide Variation in Domestication QTL Regions on Rice Chromosome 3 Suggest Lineage-Specific Selection

**DOI:** 10.1371/journal.pone.0020670

**Published:** 2011-06-06

**Authors:** Xianfa Xie, Jeanmaire Molina, Ryan Hernandez, Andy Reynolds, Adam R. Boyko, Carlos D. Bustamante, Michael D. Purugganan

**Affiliations:** 1 Center for Genomics and Systems Biology, Department of Biology, New York University, New York, New York, United States of America; 2 Department of Human Genetics, University of Chicago, Chicago, Illinois, United States of America; 3 Department of Biological Statistics and Computational Biology, Cornell University, Ithaca, New York, United States of America; 4 Department of Genetics, Stanford University, Stanford, California, United States of America; VIB & Katholieke Universiteit Leuven, Belgium

## Abstract

*Oryza sativa* or Asian cultivated rice is one of the major cereal grass species domesticated for human food use during the Neolithic. Domestication of this species from the wild grass *Oryza rufipogon* was accompanied by changes in several traits, including seed shattering, percent seed set, tillering, grain weight, and flowering time. Quantitative trait locus (QTL) mapping has identified three genomic regions in chromosome 3 that appear to be associated with these traits. We would like to study whether these regions show signatures of selection and whether the same genetic basis underlies the domestication of different rice varieties. Fragments of 88 genes spanning these three genomic regions were sequenced from multiple accessions of two major varietal groups in *O. sativa*—*indica* and *tropical japonica*—as well as the ancestral wild rice species *O. rufipogon*. In *tropical japonica*, the levels of nucleotide variation in these three QTL regions are significantly lower compared to genome-wide levels, and coalescent simulations based on a complex demographic model of rice domestication indicate that these patterns are consistent with selection. In contrast, there is no significant reduction in nucleotide diversity in the homologous regions in *indica* rice. These results suggest that there are differences in the genetic and selective basis for domestication between these two Asian rice varietal groups.

## Introduction

Crop domestication is the adaptive divergence of a plant species as a result of selection and the initial evolutionary transition from wild to human-associated cultivated environments [1], [2]. Phenotypic comparisons identify numerous traits that differ between domesticated species and their wild ancestors. In general, three classes of traits that differentiate domesticated and wild ancestral species can be defined [1]. First are domestication traits, which evolve during the initial movement of species from natural to cultivated environments. A second class is crop improvement traits, which are further phenotypic changes that have occurred after the initial domestication to human-associated cultivated environments [3]. Finally, there are crop diversification traits, which are associated with different crop varieties or cultivars adapted to different cultures or agro-ecological environments.

All three types of traits are conceptually distinct, but all can show up as differences between domesticated and wild ancestral species. It should be noted that, in principle, crop improvement traits can be difficult to separate from domestication traits. A few traits, however, are widely recognized as true domestication traits, including loss of seed shattering and change to annual life cycle [1], [2]. These traits are fixed in domesticated taxa – that is, they are phenotypes shared by all members of a domesticated crop species.

Identifying the genetic basis of domestication traits in several plant species, most especially cereal grasses, has been a major research area in the study of plant evolutionary biology [4], [5]. There have been attempts in the last few years to determine the molecular basis of cereal crop domestication, and study the nature of selection as well as other evolutionary forces associated with domestication events [4], [1]. Mapping of quantitative trait loci (QTL) associated with domestication has been a major approach in studying the genetic architecture of domestication. QTL analyses for domestication traits have been accomplished in maize [6], [7], wheat [8], pearl millet [9], foxtail millet [10] and rice [11], [12], [13], which have provided crucial information on the genetic basis of domestication. Many of these QTL studies have led to the isolation of domestication genes in various cultivated plant species [4], including the *tb1* locus that accompanies shoot architecture evolution in maize [14], and the *sh4* and *qSH1* loci that lead to loss of seed shattering in rice [15], [16].

Despite the identification of domestication trait QTLs, and in some instances domestication genes, there remain several unanswered questions surrounding the evolutionary genetics of crop domestication. First, since the putative domestication QTLs were identified using linkage mapping, it is unknown whether these mapped QTLs are indeed selected for and do not simply represent natural variation of alleles maintained by genetic drift or mutation/selection balance. Because domestication is a process of selection and adaptive evolution of cultivated species from their wild ancestor, demonstrating selection at putative domestication QTLs is a prerequisite for defining them as true domestication loci [1].

One unambiguous signature of positive selection is a “selective sweep,” which is recognized in part as significantly reduced nucleotide variation across a genomic region in proximity to a selected gene [17]. The physical extent of a sweep (whether a few hundred bp or several hundred kb) is governed by the strength of selection, time since the sweep began, and effective recombination rate between the selected site and the neighboring genomic regions. Population bottlenecks also reduce nucleotide variation levels, but this is manifested genome wide rather than the more localized decrease in polymorphisms associated with selective sweeps [18].

In several characterized domestication genes, such as maize *tb1* [14], [19], there is an unambiguous signature for positive selection, including the presence of an extended selective sweep that results in reduced nucleotide variation around the genetic target of selection [17], [1]. In other cases, however, selective sweeps have not been identified at genes that encode for presumed domestication traits. In the rice *qSW5* gene, for example, which controls variation in seed width associated with a QTL [20], population genetic analysis is still needed to characterize whether a selective sweep has indeed occurred at this gene.

A second set of issues is whether domestication within different variety groups of a crop species (for example *japonica* and *indica* rice, see below) proceeds by selection of the same genes, or whether there is selection on different genes in these different varietal groups. In recent years, it has become clear that several cereal crops, including Asian domesticated rice (*Oryza sativa* L.) and barley (*Hordeum vulgare*), appear to be comprised of genetically distinct groups [21], [22]. Comparative molecular genetic analysis of domestication QTLs or genes allows us to determine whether the same or distinct genes (or alleles) underlie evolution in these genetically distinct groups.

A final set of issues is to understand how gene flow among genetically distinct domesticated groups (*japonica* and *indica*) or even between domesticated taxa and their progenitor species affects the evolutionary dynamics of domestication. The mutant alleles of *Rc* domestication gene that lead to white pericarp in rice, for example, originated in one rice lineage and spread via introgression to another distinct *O. sativa* subspecies [23]. The importance of introgression in the spread and fixation of domestication genes during crop domestication has yet to be considered in the study of rice domestication.

To address these issues, we examine the patterns of nucleotide variation at several domestication trait QTLs in *O. sativa*, determining whether molecular diversity at these QTLs is consistent with the action of positive selection in this crop species. *O. sativa* is the world's most widely grown cereal crop species and is now a key model system in plant biology [24]. Two main rice varietal groups, *indica* and *japonica*, have been recognized since ancient China and are the most widely grown worldwide [25]. The two groups differ morphologically in grain shape and leaf color, biochemically in amylose composition, phenol reaction, and sensitivity to potassium chlorate, ecogeographically in growing environment and geographic distribution, as well as genetically in various aspects [25], [26], [27]. The *japonica* group itself is divided into the *tropical japonica* and the *temperate japonica*, the former considered to be the product of direct domestication, while the latter being a secondarily derived varietal group [25].

It has been established that *Oryza rufipogon* Griff., a species native to southeastern Asia, is the wild ancestor of domesticated rice [25], [21]. There have also been suggestions that another wild species *Oryza nivara* is the ancestor of *O. sativa* [15], although there is evidence that this species may simply be an annual ecotype of *O. rufipogon* [25], [28]. *O. rufipogon* is characterized by variable but distinctly higher levels of out-crossing, while *O. sativa* is primarily a self-fertilizing species [25]. Some genetic evidences suggest there were two domestication events for rice, with possibly separate origins for the *indica* and *japonica* groups [29], [30], [26], [21], [31], though there are other models suggesting single origin of domesticated rice [32], [33]. Early hypotheses considered that domestication of *tropical japonica* occurred in a mountainous region spanning Nepal, Assam, northern regions of Myanmar, Laos, Thailand, and the Yunnan province of southern China [21], while archaeological studies indicate that this varietal group was domesticated in the Yangtze Valley in China [34]. It was also thought that *indica* rice was independently domesticated in Ganges region of the Indian subcontinent [21], although there are suggestions that this major varietal group may have arisen in part by extensive hybridization of *tropical japonica* with either proto-*indica* or wild *O. rufipogon* [34].

In our study, we examine the molecular population genetics of genomic regions in rice that contain QTLs associated with domestication of this crop species and then compare these regions with the genome-wide data. These QTLs were identified in a large-scale mapping study between a *tropical japonica* variety (Jefferson) and a Malaysian *O. rufipogon* (IRGC 195491) [12]. The *O. rufipogon* accession used in this QTL study has been described as a weedy rice, although SSLP marker analysis clearly indicates that it is related to wild *O. rufipogon* and *O. nivara*, and is not a feral relative of domesticated rice [33].

**Figure 1:**
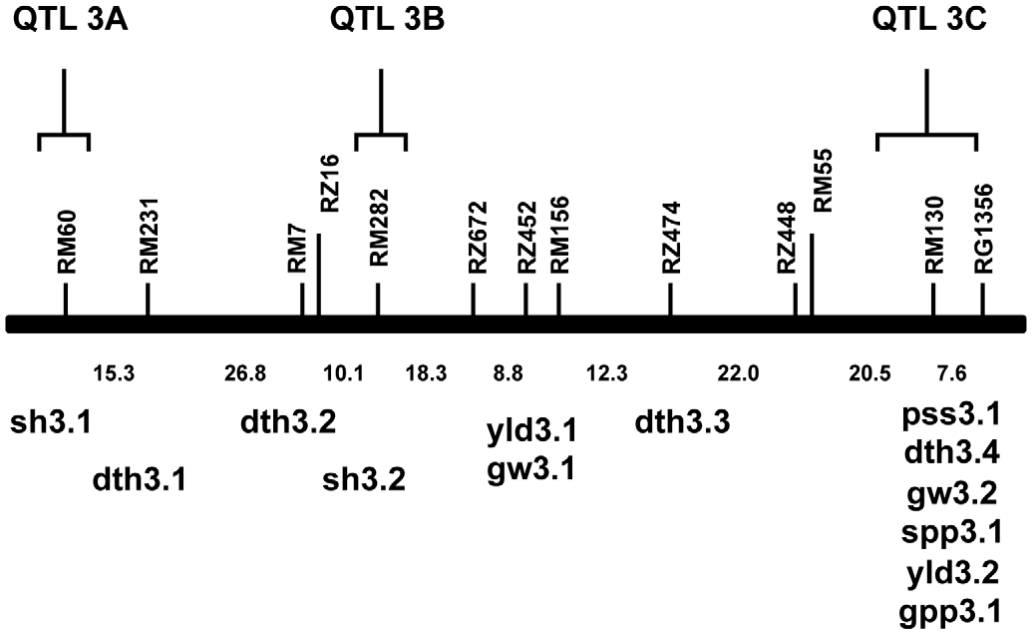
QTL map of domestication and diversification traits between *O. sativa* and *O. rufipogon*. The map is based on the study by Thomson *et al.* [12], and the regions used in our study are indicated by the square brackets. Traits associated with the QTLs are: sh, seed shattering; pss, percent seed set; dth, days to heading; gw, grain weight; spp, spikelets per panicle; yld, yield; gpp, grains per panicle.

Rice chromosome 3 was identified to contain several QTLs associated with rice domestication [12], and we decided to make this chromosome the focus of our study. Two regions at the proximal (QTL 3A) and middle (QTL 3B) of the chromosome were chosen because they were associated with loss-of-shattering, a key domestication trait. These two regions harbor the QTLs sh3.1 and sh3.2, respectively [12] (see Fig. 1). The third region at the distal end of the chromosome (QTL 3C) was chosen for analysis because multiple traits associated with domestication were localized in this one region. This region contains overlapping QTLs underlying percent seed set (pss3.1), days to heading (dth3.4), grain weight (gw3.2), the number of spikelets per panicle (spp3.1), yield (yld3.2) and the number of grains per panicle (gpp3.1) [12].

The traits that are associated with these QTLs have been implicated in the domestication of rice. We should note, however, that while QTL3A and 3B underlies a known domestication trait (e.g., loss of seed shattering), the traits associated with QTL 3C may also be considered crop improvement or diversification traits. As we indicated, telling these two types of traits apart can be difficult, and without a clear archaeological history, we can never be certain whether these traits are true domestication traits. For the purposes of this study, however, we will consider them all as domestication traits. Using re-sequencing data for gene fragments across these three putative domestication QTL regions in rice, we examine whether the levels and patterns of polymorphism in these three regions are indeed consistent with the possibility that they have experienced recent positive selection accompanying the evolution of this cultivated grass species.

## Results

### Nucleotide variation and linkage disequilibrium in three domestication QTLs

For QTL 3A, we analyzed an ∼1.05 Mb region from the proximal end of the chromosome, and in QTL 3B, we studied ∼1.9 Mb region from position 11.988 Mb to 13.863 Mb. In QTL 3C, we examined an ∼2.31 Mb region from position 32.893 Mb to 35.203 Mb. We sequenced a total of 88 gene fragments in these three QTL regions, each with an average length of 509 bp and spaced approximately 50 kb apart, totaling 44.8 kb of genomic sequence. The spacing was chosen based on previous work that indicated that linkage disequilibrium in the major rice groups extend to ∼75–150 kb [35], and that the one good example of a selective sweep in rice (in the *Waxy* gene, see [36]) is ∼260 kb in length.

**Figure 2:**
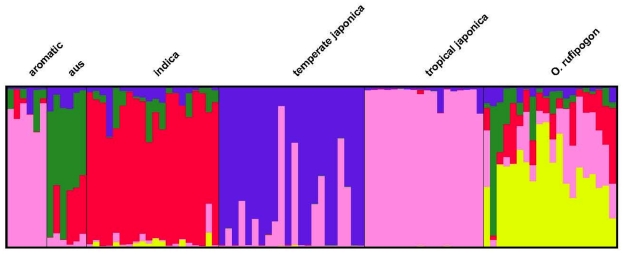
Population structure of *O. sativa* and *O. rufipogon*. It was estimated from all the loci combined from the three QTL regions. The analysis includes accessions of *temperate japonica*, *aromatic* and *aus* rices that were also sequenced for the same fragments (data not shown). The highest likelihood is found at K = 5. Vertical bars along the horizontal axis represent individual *Oryza* accessions, the proportion of ancestry that can be attributed to each cluster under K = 5 clusters is given by the length of each colored segment in a bar. The labels at the top indicate the original variety/species designation for each accession based on Garris *et al.* [26].

Previous work using genome-wide sequence tagged site (STS) data provided an indication of the genetic relationships and population structure between rice varietal groups [31]. STRUCTURE analysis using the DNA sequence data from the three domestication QTL regions is consistent with that observed using genome-wide data [31] (see Fig. 2).

**Figure 3:**
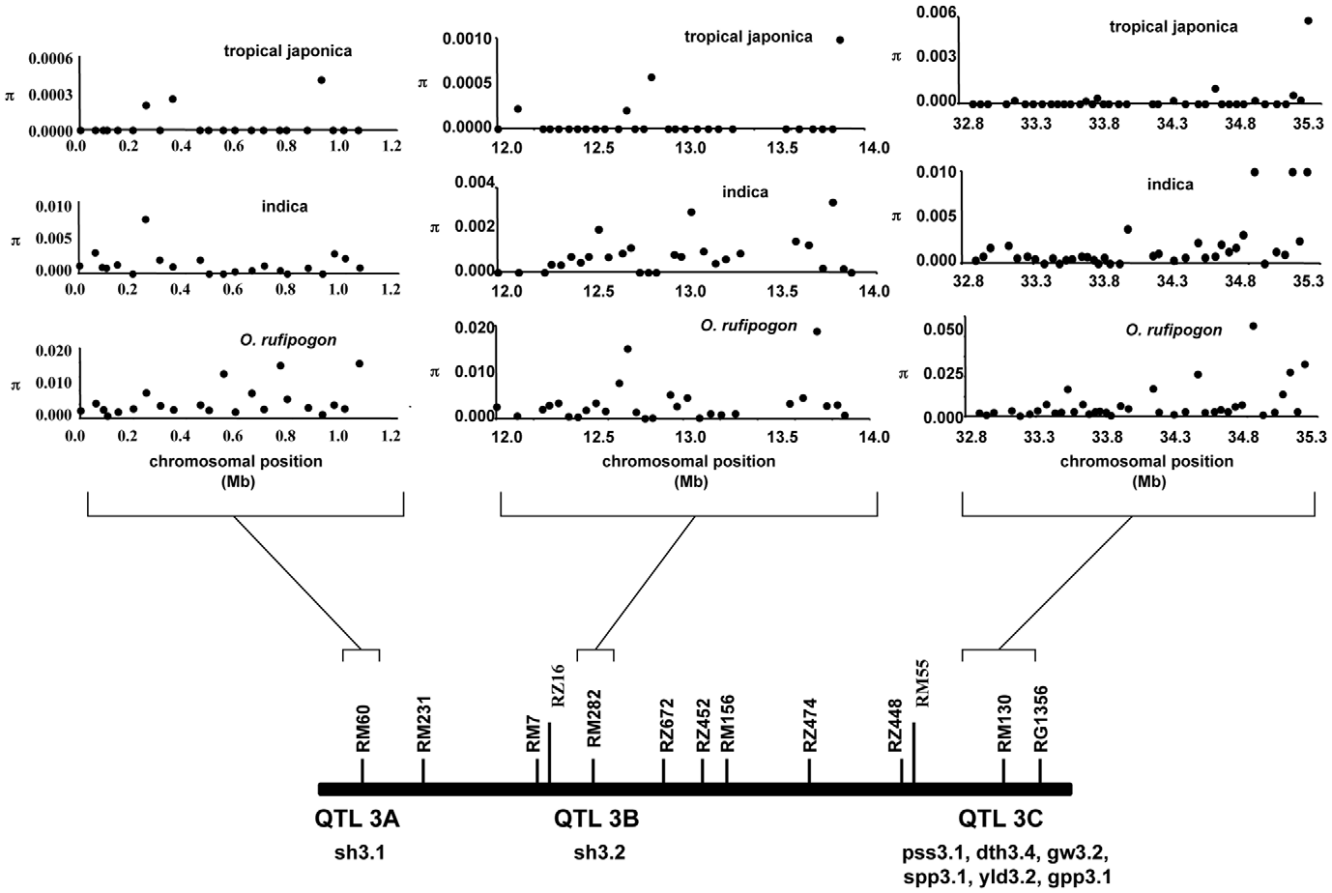
Nucleotide diversity (π) at silent sites for each gene fragment within the three QTL regions. Silent sites include both synonymous sites and noncoding sequences. Data for the two major varieties of *O. sativa* (*indica* and *tropical japonica*), as well as *O. rufipogon*, are shown.

In total, we detected 833 single nucleotide polymorphisms (SNPs) in *O. sativa* and *O. rufipogon*, of which 767 are silent site polymorphisms. The levels of silent site nucleotide variation at each of the gene fragments as well as each of the three domestication QTLs were calculated and reported for *O. rufipogon* and the two major *O. sativa* groups – *tropical japonica* and *indica*, which represent the two major domestication events in *O. sativa* (see Figure 3 and Table 1, respectively). In the domesticated rice varietal group *indica*, there are a total of 288 SNPs, with 276 at silent sites. In *tropical japonica*, there are only 37 SNPs, of which all but one are silent site changes. Mean silent site nucleotide diversity (π) across all sampled loci in *O. sativa* is approximately 0.0008 while the silent-site level of polymorphism in the wild rice species, *O. rufipogon*, is six-fold higher (π = 0.0049) (see Table 1).

**Table 1:** Silent site nucleotide diversity in domesticated rice and *O. rufipogon.*

Summary Statistics	Genomic Region	Species/Varietal group		
		*O. sativa indica*	*O. sativa* *tropical japonica*	*O. rufipogon*
**θ_w_**	**QTL 3A**	0.0013	0.00004	0.0049
	**QTL 3B**	0.0008	0.00007	0.0035
	**QTL 3C**	0.0031	0.0002	0.0064
	**STS**	0.0018	0.0015	0.0050
**π**	**QTL 3A**	0.0013	0.00007	0.0043
	**QTL 3B**	0.0008	0.0001	0.0034
	**QTL 3C**	0.0025	0.0005	0.0057
	**STS**	0.0009	0.0014	0.0050

**Figure 4:**
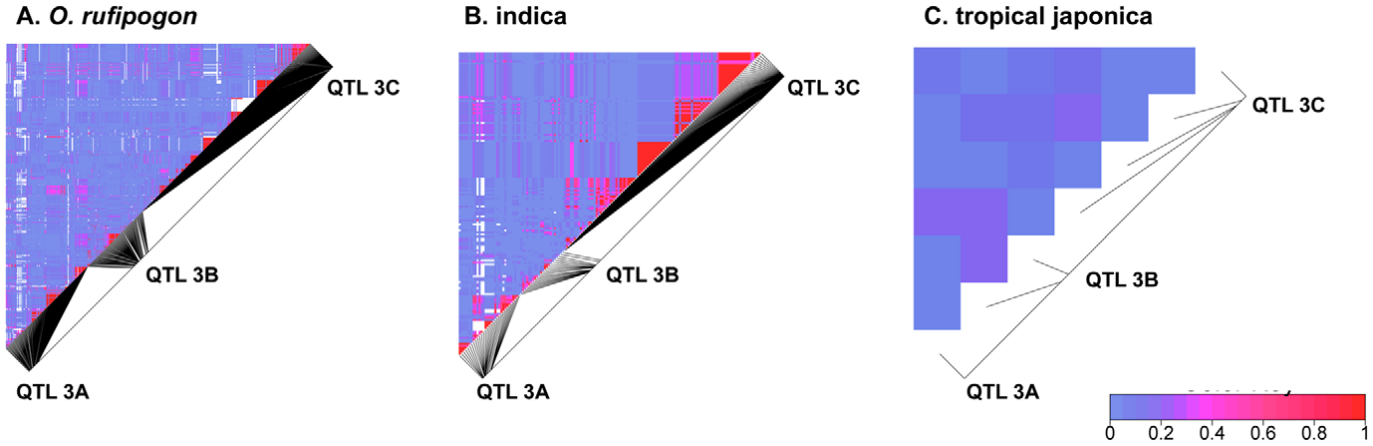
Linkage disequilibrium within and between domestication trait QTL regions. LD is measured as pairwise r^2^ [55] between SNP sites within each group, and the values are shown by different colors as indicated in the legend.

We calculated linkage disequilibrium between SNPs whose minor frequencies are greater than 10 percent within and between all three QTL regions. In the wild out-crossing species *O. rufipogon*, some linked sites within each QTL show strong disequilibrium while almost no disequilibrium is observed at sites between the three genomic regions (see Fig. 4). SNP sites in *indica* show stronger disequilibrium, compared to *O. rufipogon*, within the QTL regions (see Fig. 4). However, there are too few segregating sites remaining in *tropical japonica* to make a meaningful comparison, which suggests the selection in *tropical japonica* in these QTL regions were even stronger to have eliminated most of the polymorphism in *O. rufipogon*. The increase in LD in the domesticated rice groups have been observed in a genome-wide study [35], and is likely due to the bottleneck associated with rice domestication as well as the reduction in effective recombination in domesticated rice associated with the transition to selfing in this species.

### Levels of nucleotide variation are significantly reduced in domestication QTLs in *tropical japonica* but not *indica*

The general loss of genetic variation we observe in the three QTL regions in domesticated rice (see Table 1) is consistent with previous reports [31], [35], but the patterns of polymorphism reduction differ between the two major rice varietal groups. While the nucleotide diversity levels in *indica* at the three QTL regions are comparable to those reported previously for the genome-wide STS data [31], those in *tropical japonica* are much lower. In particular, the mean level of molecular variation in *tropical japonica* is one order of magnitude lower in the three domestication QTL regions compared to the mean genome-wide level of nucleotide diversity reported in [31].

**Figure 5:**
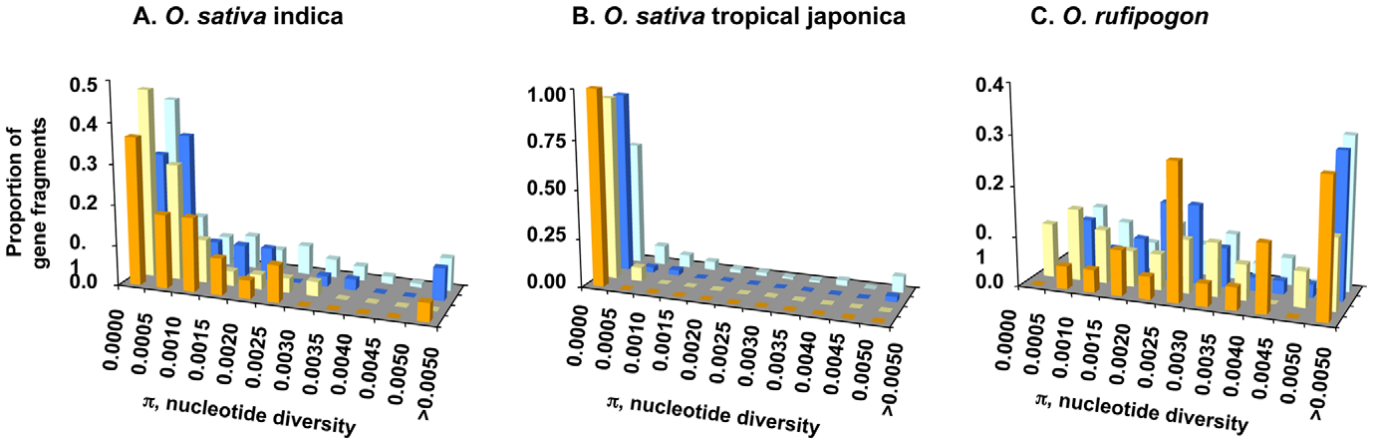
The distribution of nucleotide variation across gene fragments for three QTLs and genome-wide data. Orange, QTL 3A; yellow, QTL 3B; blue, QTL 3C; and light blue, genome-wide STS data. Note that the scale of nucleotide diversity is different in the graphs for the three different species or varietal groups.

We compared the distribution of nucleotide diversity at each of these domestication QTLs with the genome-wide distribution for the two major domesticated rice varietal groups, *indica* and *tropical japonica*, as well as the wild rice *O. rufipogon* (see Fig. 5). At QTL region 3A, the distribution of *tropical japonica* nucleotide diversity is significantly lower compared to the genome-wide distribution (Mann-Whitney Test, p<0.007). A significant reduction in nucleotide diversity at *tropical japonica* is also observed in the other two domestication trait QTLs. There is significantly lower nucleotide diversity at QTL 3B (Mann-Whitney Test, p<0.0011) and QTL 3C (Mann-Whitney Test, p<0.0101) compared to the genome-wide nucleotide diversity. Interestingly, neither *indica* rice nor the wild ancestor *O. rufipogon* shows any significant departure of nucleotide diversity distribution at all three domestication QTLs compared to the genome-wide data.

Within the three domestication trait QTL regions, we also find contiguous stretches of fragments of no polymorphism in *tropical japonica* (see Figure 3). At QTL 3A in this varietal group, two sets of large contiguous fragments of zero polymorphism are observed spanning genomic regions of ∼200 and ∼400 kb, respectively. Two extended runs of monomorphism in *tropical japonica* are also observed in both QTL 3B (∼400 and ∼900 kb in size) and 3C (∼400 and ∼350 kb in size). In contrast, the longest stretch of monomorphism in *indica* across all three domestication QTL regions is ∼250 kb in QTL 3B, which overlaps slightly with one of the monomorphic runs observed in *tropical japonica*. There are no other long tracts of low nucleotide diversity in *indica* rice or the wild rice *O. rufipogon*.

### Coalescent simulations with rice demographic model support selection in *tropical japonica*

In order to assess the statistical significance of reduced genetic variation in the three QTL regions, we need to quantify: (1) the expected levels of genetic diversity in each of the three regions under a neutral model of evolution for each of the two main subgroups (*indica* and *japonica*), and (2) the variability around this expected value due to stochasticity. In order to accomplish these two goals, we used coalescent simulations based on a complex demographic model previously inferred from genome-wide patterns of nucleotide variation [31], which considers bottlenecks at the foundation of both *indica* and *japonica* as well as migration involving *O. rufipogon*.

**Figure 6:**
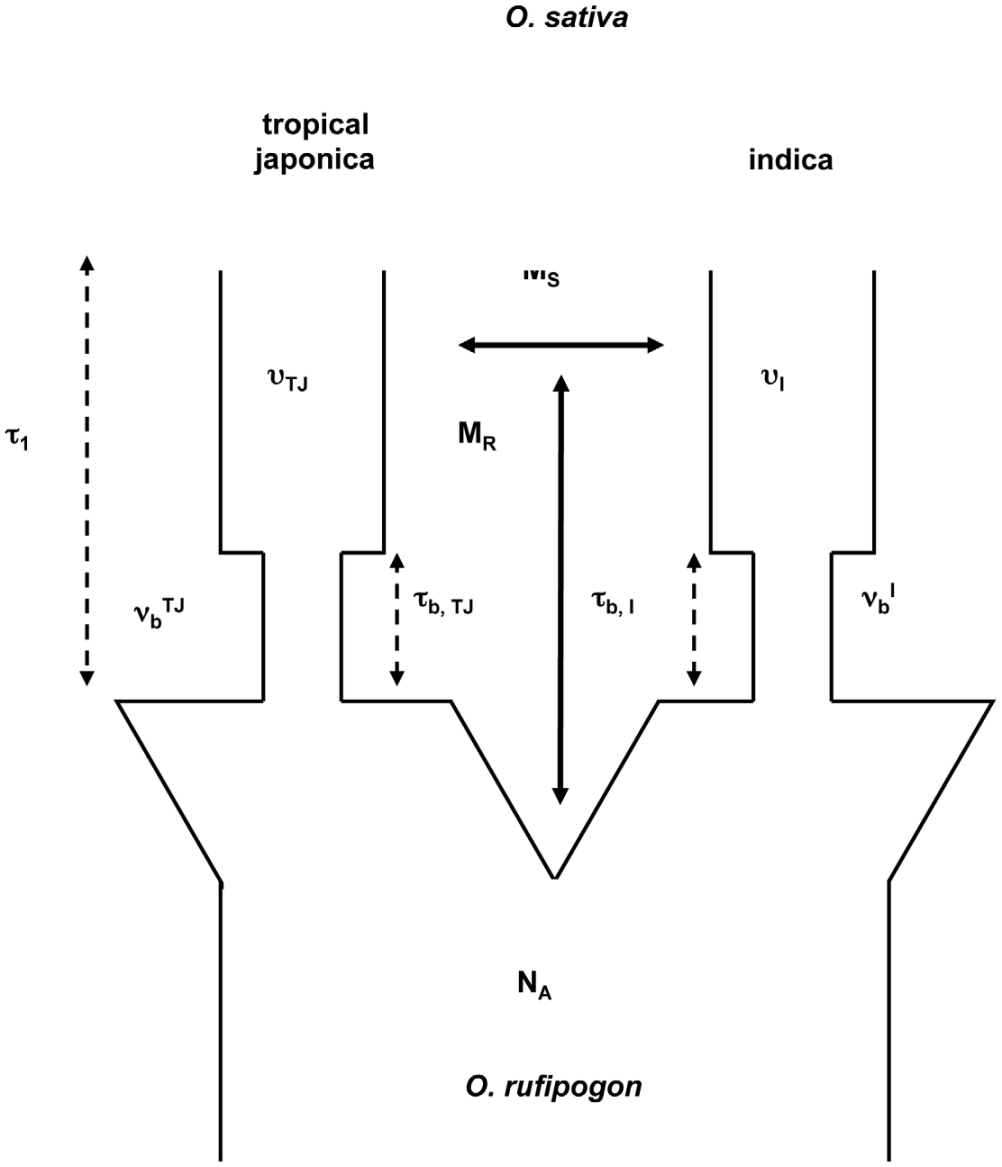
A two-origin demographic model for rice domestication. In this model, described in Caicedo *et al.* [31], the ancestral *O. rufipogon* has an ancestral population size N_A_. At τ_1_ generations ago, a bottleneck occurred with severity ν, giving rise to *tropical japonica* and *indica*. At τ_b_ generations later, we get recovery of domesticated populations to a fraction υ of the ancestral population size N_A_. The domesticated *tropical japonica* and *indica* share migrants at rate M_S_, while both domesticated groups share migrants with *O. rufipogon* at rate M_R_. For pictorial simplicity, the contemporary *O. rufipogon* population is not depicted. TJ and I indicate *O. sativa tropical japonica* and *indica*, respectively. Parameters for this model were estimated based on the unfolded site-frequency spectrum of genome-wide data [31], and were used to generate expected numbers of SNPs for each of our domestication trait QTL regions.

The low SNP levels in *tropical japonica* preclude our use of other signatures of selection such as Tajima's D or the classical site-frequency spectrum. We thus examined the observed and predicted SNP levels for each of the two domesticated rice groups, the latter of which were calculated based on the demographic model described in the Materials and Methods and in Figure 6 but informed by the observed polymorphism level of *O. rufipogon* in each QTL region. The neutral demographic model and genomic patterns of sequence variation suggests that, on average, for the number of samples drawn here (20 *O. rufipogon*, 20 *indica*, and 18 *tropical japonica*) the *O. sativa indica* sample ought to show approximately 58% of the sequence variation of the wild rice samples and the *tropical japonica* sample ought to show, on average, 41% of the variation seen in the wild ancestor. The magnitudes of these expected reductions in diversity between wild species and domesticated varietal groups are within 10% of the observed level of nucleotide diversity empirically estimated from genome-wide data [31].

Consistent with the results of the Mann-Whitney test for the difference in polymorphism level between the three QTLs and the genome-wide data, our simulation-based analysis (see Table 2) suggests too little diversity for all three regions in *tropical japonica* (p<0.001 for QTL 3A and 3B, and p<0.04 for QTL 3C). Across the three regions, the observed SNP levels in *tropical japonica* are ∼4–17 percent of the expected under the coalescent simulation. In contrast, observed diversity in *indica* ranges from ∼40–69 percent of the expected diversity based on the coalescent simulation, and do not show a significant reduction in diversity as compared to the variation one expects from the coalescent process without recombination (p<0.11 – 0.33). Given the number of multiple comparisons conducted here, it is unlikely that the *indica* deviation from expectation is biologically meaningful, while the reduced level of diversity in *tropical japonica* clearly suggests these QTL regions might have been selected in this varietal group.

**Table 2:** Observed and expected numbers of SNPs at domestication trait QTLs based on coalescent simulations.

	QTL 3A		QTL 3B		QTL 3C	
	*indica*	*japonica*	*indica*	*japonica*	*indica*	*japonica*
**Observed**	41	3	42	5	175	29
**Expected**	102.2	71.1	87.9	63.6	255.1	175.7
**Observed/Expected**	0.4	0.04	0.48	0.08	0.69	0.17
***p-value***	<0.114	<0.001***	<0.198	<0.001***	<0.330	<0.04*

*significant;

***extremely significant.

### Evolutionary relationships of domestication QTL regions in cultivated and wild species

**Figure 7:**
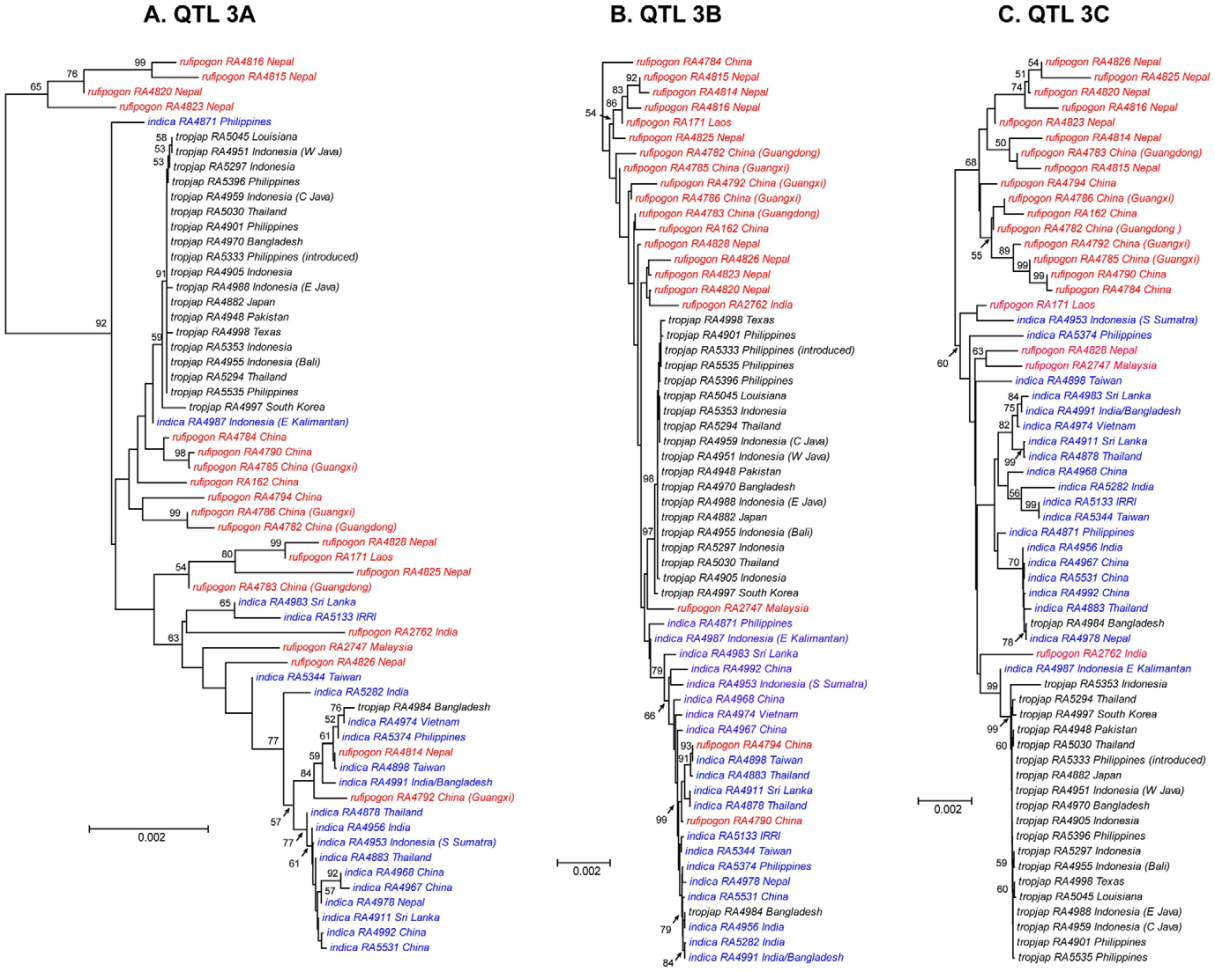
Neighbor-joining trees of the wild and domesticated rice at the domestication trait QTL regions. The accession numbers are indicated in the tree, which can be cross-referenced with Table S1.

The low levels of nucleotide diversity suggest that selective sweeps in all three QTL regions are present in *tropical japonica* but not *indica*. To examine phylogenetic relationships at these domestication QTLs, we constructed neighbor-joining trees for each genomic region spanning these QTLs (see Fig. 7). Our results show that *tropical japonica* alleles in each QTL region form a monophyletic group with moderate to high bootstrap support (67 percent for QTL 3A, 83 percent for QTL 3B, and 87 percent for QTL 3C). For QTL 3B and 3C, we find one *O. rufipogon* accession that clusters close to the *tropical japonica* clade. In QTL 3A, however, there are 11 wild rice strains that cluster with *tropical japonica* haplotypes (see Fig. 7), and eight of them are from China, consistent with an origin of this domesticated lineage in the Yangtze Valley. In contrast, none of the domestication QTL regions show the *indica* alleles forming a monophyletic clade (see Fig. 7), which again is inconsistent with a selective sweep across these genomic regions in *indica*.

## Discussion

Domestication is characterized by selection [37], which leaves its imprint on the levels and patterns of nucleotide polymorphisms within the genome [1]. Studying these molecular signatures allows us to infer the dynamics of selection as well as other evolutionary forces associated with the origin and diversification of crop species.

In rice, QTL analyses indicate that domestication traits are governed by various QTLs between *O. sativa* and *O. rufipogon* [11], [12], [13]. For the purposes of this study, we define domestication traits as either those previously shown to be associated with the origin of the cultivated species [1] or any trait fixed between the wild and domesticated species, regardless of whether this trait evolved at the origin of the cultivated species or during a post-domestication process. Most of our accessions are landraces, however, which would rule out traits (and genes) that were fixed in domesticated crop species exclusively as a result of modern breeding.

We show that the levels and patterns of nucleotide variation at three domestication trait QTLs in *O. sativa* are consistent with the recent action of selection in *tropical japonica*, as would be expected during the domestication process. The distributions of nucleotide variation among gene fragments in these QTLs are significantly different from those in a genome-wide data set, with a preponderance of low polymorphism fragments at the QTL regions (see Fig. 5). The levels of observed SNP variation are also lower in domesticated rice at these QTL regions compared to the expected values from coalescent simulations (see Table 2).

These results are similar to those observed in known selective sweeps that have previously been studied in several crop genes associated with domestication or diversification phenotypes. The best example is the maize *tb1* gene involved in the suppression of auxiliary branch formation, which has a selective sweep spanning ∼60–90-kb in length [14]. The maize *Y1* gene, involved in the yellow kernel phenotype, has a 600-kb selective sweep [38], while the rice *Waxy* gene has a 260-kb sweep associated with low-amylose rice in Northeast Asian cultivars [36]. In maize, a study analyzed 774 loci and 2–4% showed reduced variation that qualifies them as candidate domestication genes [18].

Interestingly, in our study selective sweeps are only observed in the *tropical japonica* samples but not in *indica*. This may suggest that selection at these QTL regions during domestication did not occur in *indica* rice, but was specific to *tropical japonica*. Another possibility, however, is that the *indica* alleles may comprise a “soft selective sweep.” Selective sweeps are usually considered to occur on newly arisen mutation, but soft sweeps involve selecting for an old mutation. In the case of *indica*, it may be that selection occurred on mutations that were segregating as neutral mutations for a prolonged period at appreciable frequency in the ancestral *O. rufipogon* [39], leading to a soft sweep.

There are several lines of evidence to suggest that such a soft sweep in *indica* is unlikely in this context. First, it is unclear why *tropical japonica* would experience hard sweeps (selective sweeps from newly-arisen mutations) in all 3 QTL regions and *indica* only soft sweeps, unless the genetic basis and histories in the QTL regions are markedly different. Second, the most likely result of a soft sweep would be a series of separate partial sweeps of related (but not necessarily identical) haplotypes in *indica*, which we again do not observe. Depending, however, on the specific evolutionary dynamics of such a soft sweep (e.g, a highly segregating mutation recombined in several different haplotypes coupled with widespread selection), other possible patterns of relationships may be observable, although these alternative scenarios are even less likely. Finally, while there has been discussion in the literature on the possibility of soft sweeps during domestication [39], no unambiguous cases of soft domestication sweeps have been identified, in contrast to hard sweeps for which numerous examples are known in domesticated plants and animals.

A major question in evolutionary biology is the extent to which selection in genetically distinct groups acts on different or similar genes in sculpting adaptive traits [40]. Previous studies suggest that domestication among cereal crop species may be associated with the same genes [41], [42]. Domestication traits like reduced seed shattering and increased yield have been selected in both *indica* and *japonica* rice. However, our analyses provide evidence for selection at molecular level in *tropical japonica* but not in *indica*, indicating that the genetic basis for domestication in *tropical japonica* and *indica* may differ and that separate genomic regions were subjected to selection between these two varietal groups even for the same domestication traits. This, however, is congruent with the fact that the three domestication QTLs examined in this study were identified in a mapping population between *O. rufipogon* and a *tropical japonica* cultivar of *O. sativa* [12]. A similar pattern of selection has been seen for the shattering gene *qSH1*, in which there is evidence for selection on this gene in *japonica* but not *indica* [43]. Furthermore, it appears that another gene associated with an agronomically important trait – the white pericarp *Rc* gene – was originally selected upon in *tropical japonica* and the selected allele was subsequently introgressed into *indica* [23]. Continued efforts to study the genetic architecture of domestication in rice and to examine the role of selection on genome variation and the origin of this cultivated grass species will help unravel the nature of this key evolutionary phenomenon. Moreover, since selective sweeps are a clear signature of positive selection, they can be used to identify genes associated with domestication. This novel mapping approach, which scans the genome for the selection signature of low variation across a localized genomic region [44], is known as adaptive trait locus mapping [45], hitchhiking mapping [46], or selective sweep mapping [47]. It has been successfully used in identifying the warfarin resistance locus in rats [48], and several selected loci in *Drosophila* [46] and humans [49], [50], and there is now growing interest in these methods for searching for domestication genes.

Our results suggest that one can integrate two methods to pursue these research goals - QTL mapping, which identifies specific genomic regions that harbor genes associated with specific domestication traits, and selective sweep mapping, which searches the genome for signatures of positive selection referred to as selective sweeps. By demonstrating that domestication trait QTLs do indeed harbor molecular imprints consistent with selection, it may be possible to utilize selective sweeps to further fine-map domestication genes and dissect the mechanisms that led to the origin of cultivated grass species.

## Materials and Methods

### Rice samples

The rice samples used in this study include three species: *O. sativa*, *O. rufipogon*, and *Oryza meridionalis* (see Table S1). The *O. sativa* accessions include 21 *indica* and 18 *tropical japonica* and are mainly landrace accessions, but 3 are elite cultivars. One of the *indica* accessions, POPOT-165 from Indonesia, was found by DNA sequence data to be a hybrid between *indica* and *tropical japonica* and excluded from the analyses. Most of the 20 *O. rufipogon* accessions come from China and Nepal, and a single accession of *O. meridionalis* is used as an outgroup for phylogenetic analysis.

### Gene fragments sequenced

Three domestication QTL regions on rice chromosome 3 [12] were selected in this study (see Fig. 1). The physical positions of these three QTLs were defined by identifying the flanking markers and their positions in Gramene (http://www.gramene.org). Within each of these QTLs, gene fragments of ∼500 bp in size and located ∼50 kb apart were sequenced. The sequenced fragments comprise primarily intronic sequences, and were not located in transposable elements or recent gene duplicates. A total of 88 genes were analyzed, and the number of gene fragments and associated genes within each domestication QTL are listed in Table S2.

### DNA sequencing and alignment

All primers (see Table S3) were designed using Primer3 [51] based on the *O. sativa* Nipponbare genomic sequence [52] available on Gramene. Whenever possible, the primers are designed to reside in exonic regions flanking the intron to be sequenced. All PCR primers were compared against the Nipponbare sequence to ensure that each of them uniquely recognizes the genic region to be amplified. PCR amplification and direct DNA sequencing were conducted by Cogenics (New Haven, CT, USA) as previously described [36], [31], [35]. The sequencing error rate was assessed as previously described [31], which revealed an error rate of less than 0.01%.

Base-pair calling, quality score assignment, and construction of sequence contigs were carried out using the Phred and Phrap programs (CodonCode), and sequence alignment and editing were carried out with BioLign Version 4.0.5.1 (Tom Hall, North Carolina State University, Raleigh, North Carolina). Single nucleotide polymorphisms (SNPs) were identified as mutational differences between sequenced alleles. Heterozygous sites and insertion/deletions were identified with the aid of Polyphred (Deborah Dickerson, University of Washington, Seattle, Washington) and manually confirmed by visually inspecting chromatograms. Primer sequences were removed from the alignments for final analysis. All sequences are deposited in Genbank with accession numbers FJ015311–FJ023534.

### Analysis of nucleotide diversity

Population genetic structure at the three QTL regions was assessed using STRUCTURE 2.2 [53]. Simulations were run with a linkage model and allele frequencies being independent among populations. Five replicates at each value of K (population number, from 2–9) were carried out, and each run had a burn-in length and a run length of 100,000 iterations.

Nucleotide diversity (π) and Watterson's theta θ_W_ [54] were calculated for individual domesticated rice varieties, as well as for *O. rufipogon*. The average nucleotide diversity (π) in each QTL was compared to genome-wide STS data [31] using a non-parametric Mann-Whitney test. The ratio of θ_W_ for each domesticated rice group and that for the wild *O. rufipogon* for each fragment was also calculated and compared with nucleotide variation for gene fragments across the genome [31].

Pairwise SNP linkage disequilibrium within each QTL region was assessed with r^2^ [55], implemented using the program TASSEL (http://www.maizegenetics.net). All sites where the minor allele frequency was <10%, or where more than two alleles at a SNP site were excluded. Accessions were also excluded from the analyses if they have missing data or gaps in one or both of the SNP sites. We treated heterozygous SNPs as previously described [35]. Heterozygous sites are rare in *O. sativa* individuals, but more frequent in *O. rufipogon* SNP genotypes. We excluded an individual from analysis if it was heterozygous at both SNP sites in a pair so that only unambiguous haplotypes were used in the analysis. In *O. rufipogon*, the majority of the SNP pairs containing individuals with double heterozygotes had only a single doubly heterozygous individual to exclude. To generate a graphical display of pairwise LD measurements, a script written by Shin *et al.* [56] was run in R.

### Coalescent simulations

Coalescent theory allows us to trace the evolution of alleles in a population sample to a single ancestral copy, and provides a framework to test whether SNP data from a sample is consistent with neutral evolution [57]. We assessed the statistical significance of reduced genetic variation in the three QTL regions using coalescent simulations based on a demographic model previously inferred from genome-wide patterns of nucleotide variation [31]. Maximum composite-likelihood parameters for this model had been previously estimated using the joint site-frequency spectrum for the genome-wide data [31].

The model has the following features: The ancestral species *O*. *rufipogon* is assumed to have a constant population size, N_A_, which is a reasonably good fit for the observed genome-wide site-frequency spectrum. Based on a previous study [31], we assume that *indica* and *tropical japonica* split simultaneously from *O. rufipogon* and formed separate populations 4*N_A_*0.1 generations ago with each undergoing a bottleneck and then post-bottleneck growth. The bottleneck model for the *indica* lineage is as follows (looking back in time): from present to 4*N_A_*0.04 generations ago, we set Ne for *indica*  = 0.27*N_A_ to model post-bottleneck growth; from 4*N_A_*0.04 to 4*N_A_*0.1 generations ago, we set Ne  = 0.0055*N_A_ corresponding to the domestication bottleneck; and prior to this time, Ne = N_A_, to reflect common ancestry with *O. rufipogon*. For *tropical japonica*, the corresponding parameters are as follow: from present to 4*N_A_*0.038104 generations ago (post-bottleneck growth in *japonica*), we set Ne  = 0.12*N_A_; from 4*N_A_*0.038104 to 4*N_A_*0.1 generations ago (domestication bottleneck in *japonica*), Ne  = 0.0055*N_A_; and, prior to 0.01*4*N_A_, Ne for *japonica*  =  N_A_. It is important to note that this model allows for migration among the three populations. Specifically, in each generation, an average of 7 migrants enter the *O. rufipogon* population (equally from the other two populations), based on results from the previous study [31]. Both *indica* and *tropical japonica* receive migrants at a rate proportional to their relative population size at each generation with 0.0385 migrants during the bottleneck, 1.89 and 0.84 migrants entering *indica* and *tropical japonica*, respectively, after the bottleneck.

In the simulations we also accounted for the possible impact of local variation in mutation rate scaled on population size. Specifically, for each of the three QTL regions we estimated the baseline mutation rate for each of the three QTL regions using the *O. rufipogon* sequence. Previous work and the observed distribution of variable nucleotide frequencies in the present study suggests that *O. rufipogon* demography is accurately described by the standard neutral model so that Watterson's estimate of the mutation rate (θ_W_) is an appropriate summary statistic from which to estimate this quantity [31]. We simulated 1,000 replicate data sets for each QTL region separately using the demographic model described above. In order to assess significance, we tallied the number of simulated data sets that show as little as or less diversity than the observed for each subgroup and for each QTL region.

### Phylogenetic analysis

Fragments within each QTL region were concatenated, and neighbor joining analyses using an improved algorithm [58] were performed in PAUP v4.0 beta Win [59] on each concatenated dataset with K2P distance correction and gamma setting. Negative branch lengths were prohibited. Strict consensus trees were rooted using the outgroup species *O. meridionalis*. Bootstrap (BS) support values were obtained in PAUP using 500 replicates applying the NJ search option. Tree files were viewed using the program of FigTree v1.2.2 by A. Rambaut (http://tree.bio.ed.ac.uk/software/figtree/).

## Supporting Information

Table S1 **Rice accessions used in this study.**
(XLS)

Table S2 **Genes associated with sequenced fragments at each QTL and their function.**
(XLS)

Table S3 **Primers used to amplify the gene fragments used in this study.**
(XLS)
